# Drug treatment and prevention of malaria in pregnancy: a critical review of the guidelines

**DOI:** 10.1186/s12936-020-03565-2

**Published:** 2021-01-23

**Authors:** Khalid A. J. Al Khaja, Reginald P. Sequeira

**Affiliations:** Department of Pharmacology & Therapeutics, College of Medicine & Medical Sciences, Arabian Gulf University, P.O. Box 22979, Manama, Kingdom of Bahrain

**Keywords:** Treatment guidelines, National guidelines, WHO, Recent trends, Malaria, Pregnancy, Harmonization

## Abstract

**Background:**

Malaria caused by *Plasmodium falciparum* in pregnancy can result in adverse maternal and fetal sequelae. This review evaluated the adherence of the national guidelines drawn from World Health Organization (WHO) regions, Africa, Eastern Mediterranean, Southeast Asia, and Western Pacific, to the WHO recommendations on drug treatment and prevention of chloroquine-resistant falciparum malaria in pregnant women.

**Methods:**

Thirty-five updated national guidelines and the President’s Malaria Initiative (PMI), available in English language, were reviewed. The primary outcome measures were the first-line anti-malarial treatment protocols adopted by national guidelines for uncomplicated and complicated falciparum malaria infections in early (first) and late (second and third) trimesters of pregnancy. The strategy of intermittent preventive treatment of malaria in pregnancy (IPTp) with sulfadoxine-pyrimethamine (SP) was also addressed.

**Results:**

This review evaluated the treatment and prevention of falciparum malaria in pregnancy in 35 national guidelines/PMI-Malaria Operational Plans (MOP) reports out of 95 malaria-endemic countries. Of the 35 national guidelines, 10 (28.6%) recommend oral quinine plus clindamycin as first-line treatment for uncomplicated malaria in the first trimester. As the first-line option, artemether–lumefantrine, an artemisinin-based combination therapy, is adopted by 26 (74.3%) of the guidelines for treating uncomplicated or complicated malaria in the second and third trimesters. Intravenous artesunate is approved by 18 (51.4%) and 31 (88.6%) guidelines for treating complicated malaria during early and late pregnancy, respectively. Of the 23 national guidelines that recommend IPTp-SP strategy, 8 (34.8%) are not explicit about directly observed therapy requirements, and three-quarters, 17 (73.9%), do not specify contra-indication of SP in human immunodeficiency virus (HIV)-infected pregnant women receiving cotrimoxazole prophylaxis. Most of the guidelines (18/23; 78.3%) state the recommended folic acid dose.

**Conclusion:**

Several national guidelines and PMI reports require update revisions to harmonize with international guidelines and emergent trends in managing falciparum malaria in pregnancy. National guidelines and those of donor agencies should comply with those of WHO guideline recommendations although local conditions and delayed guideline updates may call for deviations from WHO evidence-based guidelines.

## Background

Malaria caused by *Plasmodium falciparum* in pregnancy can result in adverse maternal and fetal sequelae [1]. Pregnant women are at increased risk of maternal anaemia, placental malaria and death [1–3]. However, stillbirth, premature birth, intra-uterine growth restriction, and low birth weight (LBW) are poor fetal/newborn pregnancy outcomes [1–3]. Placental malaria results from the sequestration of infected red blood cells (RBCs) in the intervillous spaces of the placenta and binding of parasites to surface chondroitin sulfate-A (CSA). Furthermore, the recruitment of macrophages and pro-inflammatory cytokines in response to parasite-infected RBCs contributes to the thickening of the placental basement membrane, which interferes with the maternal and fetal exchange mechanisms, leading to poor outcomes [4, 5]. Immunity to placental malaria is acquired during the first and subsequent pregnancies as women develop antibodies against parasite-binding sites to CSA: it blocks *P. falciparum* sequestration into intervillous spaces, and enhances the opsonic clearance of parasitized erythrocytes [4–7].

The symptoms and sequelae of malaria in pregnancy vary according to the severity of transmission of malaria and levels of immunity acquired by individuals [1]. For areas with moderate-to-high (stable) malaria transmission (areas characterized by steady prevalence pattern, with little variation from 1 year to another and affected population often has high levels of immunity), pregnant women appear to have high acquired immunity; thus, they are paucisymptomatic during infection [1, 6]. Prompt diagnosis and treatment of malaria infection, whether symptomatic or paucisymptomatic, are required to prevent maternal anaemia and LBW of the newborn [1, 6]. Moreover, malaria prevention and treatment are essential components of antenatal care, especially in regions where the malaria parasite is endemic, as in sub-Saharan Africa (SSA) [1, 4]. The World Health Organization (WHO) has recommended the intermittent preventive treatment in pregnancy (IPTp) strategy in which a single dose of three tablets of single-pill combination (SPC) of sulfadoxine-pyrimethamine (SP) is administered integral to antenatal care service [8, 9]. This strategy mitigates or prevents malaria-related adverse maternal and fetal outcomes. The IPTp-SP is implemented in pregnant women starting as early as possible in the second trimester, with SP administered at monthly intervals up to the time of delivery. IPTp-SP should be administered under directly observed therapy (DOT) along with folic acid dose reduction (400 µg daily), usually with iron for prevention of maternal anaemia. IPTp-SP strategy is contra-indicated in pregnant women who are human immunodeficiency virus (HIV)-positive, receiving cotrimoxazole prophylaxis in malaria-endemic regions of Africa.

In areas with low (unstable) malaria transmission (areas characterized by considerable variation in incidence pattern from 1 year to another and the population usually has little immunity), pregnant women appear to have low antibody-mediated immunity to malaria infection: they are more likely to develop severe malaria syndrome with cerebral malaria, hypoglycaemia and respiratory distress [1, 10]. In such regions, malaria is associated with a greater risk of spontaneous abortion, stillbirth, prematurity, and LBW [1, 6, 7]. Both *primigravidae* and *multigravidae* are equally susceptible [10]. IPTp-SP strategy cannot be implemented in low malaria transmission areas in the Greater Mekong Sub-region (GMS) and some African countries because of high drug resistance [1].

The rational use of anti-malarial drugs for treating of falciparum malaria in pregnancy in each country is determined by several variables that include therapeutic efficacy of drugs against *P. falciparum*, maternal and fetal adverse events, safety concerns, and treatment cost-effectiveness [1]. Only anti-malarials with a proven safety profile supported by robust clinical evidence are used to treat malaria during pregnancy [1]. Physiological changes associated with pregnancy and their potential effects on anti-malarial pharmacokinetics are important considerations [11]. WHO-recommended, evidence-based, malaria treatment protocols that can be safely used in pregnancy [1] are presented in Table 1. Anti-malarials, such as primaquine, tafenoquine (for management of recurrent vivax/ovale malaria), and tetracyclines, are contra-indicated in pregnancy.

**Table 1:** Anti-malarial drug treatment recommended by 2015 WHO guidelines for falciparum malaria in pregnancy

Malaria severity/trimesters	First-line treatment	Dosage/frequency	Alternative options/implementation strategy issues
Uncomplicated malaria in first trimester	Quinine (Q) + Clindamycin (C), oral	Q: 10 mg/kg twice a day for 7 days C: 10 mg/kg twice a day for 7 days	Q monotherapy if C is not available or ACT or oral artesunate + C is an alternative if Q + C if not available or fails
Uncomplicated and complicated malaria in second and third trimesters	*Single pill combination (SPC) of ACT*		With respect to AL, it should be administered along with milk or fat-rich meal to enhance its oral bioavailability
	Artemether + Lumefantrine (AL)	4 tabs (20 mg + 120 mg) given at 0, 8, 24, 36, 48, and 60 h over 3 consecutive days	
	Artesunate + Amodiaquine (AS–AQ)	2 tabs (100 mg + 270 mg) given once daily for 3 consecutive days	
	Dihydroartemisinin + Piperaquine (DHA–PPQ)	4 tabs (40 mg + 320 mg) given once daily for 3 consecutive days	
Complicated malaria during all trimesters	Parenteral artesunate (AS)	2.4 mg/kg at 0, 12, 24, and 48 h	If AS is unavailable, intramuscular artemether should be given, and if this is unavailable, then parenteral Q should be started immediately until AS is obtained. Following parenteral AS, treatment should be completed with a full treatment course of oral Q + C for complicated malaria in first trimester or oral ACT for uncomplicated and complicated malaria in 2nd and 3rd trimesters of pregnancy
Intermittent preventive treatment in pregnancy (IPTp) in moderate-to-high malaria transmission areas^a^	SPC of sulfadoxine-pyrimethamine (SP)	3 tabs (500 mg + 25 mg) given at every scheduled antenatal visit from 2nd trimester at least 1 month apart up to delivery	Should be given as directly observed therapy (DOT) along with folic dose reduction (400 µg daily). IPTp is implemented in pregnant women starting as early as possible at beginning of 2nd trimester around 13th week of gestation with SP administered at monthly intervals up to the time of delivery and is contra-indicated in women co-infected with HIV on cotrimoxazole prophylaxis

^a^ Areas characterized by steady prevalence pattern, with little variation from 1year to another and affected population often has high levels of immunity

Parenteral stands for intravenous

Malaria caused *by Plasmodium vivax* in Asia–Pacific regions is, although considered benign, associated with adverse pregnancy outcomes and requires prompt and effective management with parenteral artesunate as for severe falciparum malaria [1, 12]. Following parenteral artesunate, treatment can be completed with the full treatment course of oral artemisinin-based combination therapy (ACT) (in countries with chloroquine-resistant *P. vivax*) or chloroquine (in countries where chloroquine is the treatment of choice) [1]. The eradication of hepatic-stage parasites with primaquine or tafenoquine is deferred until after delivery. Malaria caused by *Plasmodium knowlesi* appears to be more severe than infections by other non-falciparum species and is treated as for falciparum infection [13].

This review aims to evaluate critically the degree to which national guidelines comply with WHO recommendations [1] to treat and prevent chloroquine-resistant *P. falciparum* in pregnancy, specifically to include:

- uncomplicated malaria in the first trimester;
- uncomplicated and complicated malaria in the second and third trimesters;
- complicated malaria during all trimesters;
- chemoprophylaxis of malaria in pregnancy in moderate-to-high transmission areas.

In this review, the focus is on falciparum malaria and not on other species common in some of the countries reviewed in view of the magnitude of the problem due to implications on perinatal morbidity and mortality, emergent anti-malarial resistance and updates on drug therapy recommendations.

## Methods

### Literature survey

National and international malaria treatment guidelines were retrieved using PubMed Medical Subject Heading terms: “guidelines” and “malaria”. The Worldwide Web via Google was used with the following phrases: malaria treatment guidelines followed by the country name (e.g., Angola, Thailand) or organizations such as WHO, US Centers for Disease Control and Prevention (US CDC) and President’s Malaria Initiatives (PMI)-Malaria Operational Plans (MOP). The data analysis was limited to publications, literature and reports from December 2012 onwards about 2 years after the release of the second edition of WHO Guidelines for Treatment of Malaria [14]. It was assumed that 2 years would suffice for guideline dissemination and for countries to adopt WHO Guidelines.

### Inclusion and exclusion criteria

Updated national guidelines emerged 2012 onwards and PMI-MOP reports for 2018–2019 published in English were included. Based on WHO regional classification [15], countries from Africa, Eastern Mediterranean, Southeast Asia, and Western Pacific regions known to have the highest estimated malaria prevalence were included. National guidelines/PMI-MOP reports written in languages other than English, guidelines published before 2012, and Pan-American Health Organization (PAHO) countries were excluded.

Except for malaria chemoprevention in pregnancy, there was no significant difference between the 3rd [1] and 2nd [14]. WHO Guidelines recommendations reported with regard to first-line treatment in uncomplicated and complicated falciparum malaria in all trimesters of pregnancy (Table 2). Since national guidelines in Malaysia (2013) and national guidelines in both India and Sri Lanka (2014) were published 1–2 years ahead of the 2015 WHO Guidelines [1] and 3–4 years after publication of the 2010 WHO Guidelines [14], both WHO Guidelines editions were used in this review (Table 2).

**Table 2:** Treatment and chemoprevention of falciparum malaria in pregnancy: international and national guidelines recommendations

Guidelines	Treatment of chloroquine-resistant *P. falciparum* in pregnancy				Malaria chemoprevention in pregnancy		
	Uncomplicated		Complicated (severe)		IPTp-SP	Folic acid/day	Concurrent use of CTX + IPTp-SP
	1st trimester	2nd and 3rd trimester	1st trimester	2nd and 3rd trimester			
WHO 2010 [14]	Oral Q + C for 7 days	ACT (excluding DHA–PPQ)	Parenteral AS; a full course of oral Q + C as F-OT	Parenteral AS; a full course of oral ACT as F-OT	Not included	Not specified	Contra-indicated
WHO 2015 [1]	Oral Q + C for 7 days	ACT (including DHA–PPQ)	Parenteral AS; a full course of oral Q + C as F-OT	Parenteral AS; a full course of oral ACT as F-OT	Recommended	400 µg	Contra-indicated
CDC 2019 [56]	Oral MQ/Q + C for 3 or 7 days^a^	MQ/A-L/Q + C	Parenteral AS; a full course of MQ/Q + C as F-OT	Parenteral AS; a full course of oral A-L/Q + C as F-OT	Not included	Not specified	Not included
Africa							
Angola^§^ [54] 2019	Oral Q for 7 days	AL	Parenteral AS^†^	Parenteral AS^‡^	Implemented**	5000 µg	Not stated
Benin^§^ [54] 2018	Oral Q for 7 days	AL/AS–AQ	Parenteral Q^†^	Parenteral Q^‡^	Implemented**	400 µg	Not stated
Burkina Faso^§^ [54] 2019	Oral Q for 7 days	AL	Parenteral Q^†^	Parenteral AS^‡^	Implemented*	250 µg	Contra-indicated
Cameroon^§^ [54] 2019	Parenteral Q	Parenteral AS	Parenteral Q	Parenteral AS	Implemented*	Up to 5000 µg	Contra-indicated
DRC (Congo)^§^ [54] 2018	Oral Q + C for 7 days	AS–AQ/AL	Parenteral Q^†^	Parenteral AS^‡^	Implemented *	400 µg	Contra-indicated
Côte d’Ivoire^§^ [54] 2019	Oral Q for 7 days	AS–AQ/AL/DHA–PPQ	Parenteral Q^†^	Parenteral Q^‡^	Implemented*	400 µg	Contra-indicated
Ethiopia^§^ [54] 2019	Oral Q for 7 days	AL	Parenteral AS^†^	Parenteral AS^‡^	Not implemented	–	–
Ghana^§^ 2018	Oral Q for 7 days	AS–AQ/AL	Parenteral Q^†^	Parenteral AS^‡^	Implemented**	400 µg	Not stated
Guinea^§^ [54] 2018	Oral Q for 7 days	AL/AS–AQ	Parenteral Q^†^	Parenteral AS^‡^	Implemented*	250 µg	Not stated
Kenya^§^ [54] 2018	Oral Q for 7 days	AL	Parenteral AS^†^	Parenteral AS^‡^	Implemented*	400 µg	Not stated
Liberia^§^ [54] 2019	Oral Q for 7 days	AS–AQ/AL	Parenteral AS^†^	Parenteral AS^‡^	Implemented**	250–400 µg	Not stated
Madagascar^§^ [54] 2018	Oral Q for 7 days	AS–AQ	Parenteral AS^†^	Parenteral AS^‡^	Implemented**	400 µg	Not stated
Malawi^§^ [54] 2018	Oral Q + C for 7 days	AL	Parenteral Q^†^	Parenteral AS^‡^	Implemented*	400 µg	Not stated
Mali^§^ [54] 2018	Oral Q for 7 days	AL	Parenteral AS^†^	Parenteral AS^‡^	Implemented*	400 µg	Not stated
Mozambique^§^ [54] 2019	Oral Q for 7 days	AL	Parenteral Q^†^	Parenteral AS^‡^	Implemented*	1000 µg	Not stated
Niger^§^ [54] 2019	Oral Q for 7 days	ACT	Parenteral Q^†^	Parenteral AS^‡^	Implemented*	Not specified	Not stated
Nigeria^§^ [54] 2019	Oral Q + C for 7 days	AL/AS–AQ	Parenteral AS^†^	Parenteral AS^‡^	Implemented*	Not specified	Not stated
Rwanda^§^ [54] 2019	Oral Q for 7 days	AL	Parenteral AS^†^	Parenteral AS^‡^	Discontinued	–	Not stated
Senegal^§^ [54] 2018	Oral Q for 7 days	AL/AS–AQ/DHA–PPQ	Parenteral AS^†^	Parenteral AS^‡^	Implemented*	400 µg	Not stated
Sierra Leone^§^ [54] 2019	Oral Q + C for 7 days	AL	Parenteral AS^†^	Parenteral AS^‡^	Implemented**	400 µg	Not stated
South Africa [46] 2019	Oral Q + C for 7 days	AL	Parenteral AS^†^	Parenteral AS^‡^	Not implemented	–	–
Tanzania^§^ [54] 2019	Oral Q for 7 days	AL	Parenteral Q^†^	Parenteral AS^‡^	Implemented*	250 µg	Not stated
Uganda^§^ [54] 2019	Oral Q for 7 days	AL	Parenteral Q^†^	Parenteral AS^‡^	Implemented*	400 µg	Contraindicated
Zambia^§^ [54] 2019	Oral Q for 7 days	AL	Parenteral Q^†^	Parenteral AS^‡^	Implemented**	Low dose (not specified)	Not stated
Zimbabwe^§^ [54] 2018	Oral Q + C for 7 days	AL	Oral Q	Oral Q	Implemented*	400 µg	Contraindicated
Eastern Mediterranean							
Saudi Arabia [45] 2018	Oral Q + C for 7 days	AL/AS–MQ	Parenteral AS^†^	Parenteral AS^‡^	NA	–	Contraindicated***
Somalia^§¶^ [60] 2016	Oral Q for 7 days/AL^$^	AL	Parenteral AS^†^	Parenteral AS^‡^	Implemented**	250 µg	Not stated
Sudan^§¶^ [59] 2017	Oral Q for 7 days	AL/DHA–PPQ	Parenteral Q^†^	Parenteral Q/AS^‡^	Not implemented	–	–
South-East Asia							
Bangladesh [72] 2016	AL/AS–AQ/oral Q + C	AL/AS–AQ	Parenteral AS/Q^†^	Parenteral AS^‡^	NA	NA	–
Burma^#^ [54] 2018	Oral Q + C for 7 days	AL	Parenteral AS^†^	Parenteral AS^‡^	NA^●^	NA	–
India[73] 2014	Oral Q for 7 days	AL	Parenteral Q/AS^†^	Parenteral AS^‡^	NA	NA	–
Sri Lanka [55] 2014	Oral Q + C for 7 days	AL	Parenteral Q^†^	Parenteral AS/Q^‡^	NA	NA	–
Thailand^#^ [54] 2018	Oral Q + C for 7 days	DHA–PPQ	Parenteral AS^†^	Parenteral AS^‡^	NA^●^	NA	–
Western Pacific							
Cambodia^#^ [54] 2018	Oral Q for 7 days	DHA–PPQ/AS–MQ	Parenteral AS^†^	Parenteral AS^‡^	NA^●^	NA	–
Malaysia [74] 2013	Oral Q for 7 days	AL/AS–MQ	Parenteral AS^†^	Parenteral AS^‡^	NA	NA	–

A: artemether; ACT: artemisinin-based combination therapy; AL: artemether-lumefantrine; AS: artesunate; AS–AQ: artesunate-amodiaquine; AS–MQ: artesunate-mefloquine; C: clindamycin; CTX: cotrimoxazole; DHA–PPQ: dihydroartemisinin-piperaquine; DRC: Democratic Republic of the Congo; F-OT: follow-on treatment; IPTp-SP: intermittent preventive treatment in pregnancy with sulfadoxine-pyrimethamine; MQ: mefloquine; Q: quinine; SP: sulfadoxine-pyrimethamine; SPC: single-pill combination

NA not applicable

NA^●^ it is not part of a national policy due to potential low (unstable) transmission or prevalence of malaria/no funding allocated for malaria in pregnancy by PMI/resistance to sulfadoxine-pyrimethamine (SP)

^a^ Quinine given for 3 days, except for infections acquired in Southeast Asia where 7 days of treatment is required

* Under directly observed therapy

** Directly observed therapy is not undertaken

*** ACT regimen containing SP should not be given to HIV-infected pregnant patients receiving cotrimoxazole as this increases the risk of sulfonamide-induced adverse reactions

^†^ Following parenteral artesunate/quinine at least for 24 h and when the patient becomes able to take oral therapy, a full treatment course of anti-malarial(s) recommended for uncomplicated malaria during the 1st trimester (quinine alone or quinine + clindamycin) is given as follow-on treatment

^‡^ Following parenteral artesunate/quinine for at least 24 h and when the patient can take oral therapy, a full course of ACT recommended for uncomplicated malaria during the 2nd and 3rd trimester is given as follow-on treatment

^¶^ Geographically sub-Saharan African countries although these are members of the Arab League as well

^#^ Greater Mekong Sub-region; (President’s Malaria Initiative-supported countries)

^§^ Sub-Saharan African countries (President’s Malaria Initiative-supported countries)

^$^ AL is given at a dose and duration similar to that of adult patients (see Table 3)

– Not included in the relevant guidelines or the operational malaria plan

### Outcome measures

According to WHO recommendations on treatment of chloroquine-resistant falciparum malaria in pregnant women, the primary outcome measures evaluated were: (1) first-line drugs for treating either uncomplicated or complicated (severe) malaria infection during any trimesters of pregnancy; (2) IPTp-strategy with SP combination, guidance on folic acid supplementation and concomitant IPTp-SP with cotrimoxazole in HIV-positive pregnant women.

### Validity assessment

The authors independently carried out an assessment of outcome measures in a non-blinded standardized manner. Any discrepancy was resolved in consultation with another clinical pharmacologist.

### PMI definition

PMI is a US Government initiative, one of the largest international sources of funding to control and eradicate malaria in PMI-supported countries. PMI works in partnership with host-country governments in SSA and GMS to: (a) provide effective therapeutic and prophylactic intervention to those at risk of malaria; (b) develop annual MOPs; and, (c) complement and expand malaria monitoring and evaluation strategies [16].

## Results

A total of 38 international and national guidelines and updated PMI-MOP reports fulfilled the inclusion criteria. WHO region-wide breakdown of the 35 national guidelines/reports was as follows: SSA: 25, Eastern Mediterranean: 3, Southeast Asia: 5, Western Pacific region: 2.

### Uncomplicated falciparum malaria in first trimester

Contrary to WHO recommendations [1], oral quinine monotherapy instead of quinine plus clindamycin regimen was adopted by 23/35 (65.7%) guidelines/reports as first-line treatment for uncomplicated falciparum malaria in the first semester of pregnancy (Table 2).

### Uncomplicated and complicated falciparum malaria in second and third trimesters

As first-line treatment, artemether-lumefantrine (AL), an SPC, was the most common artemisinin-based combination recommended by 26/35 (74.3%) guidelines for treating uncomplicated malaria in second and third trimesters, and as the preferred option for completing treatment following parenteral artesunate/quinine for complicated malaria in second and third trimesters. The proportion of national guidelines/PMI reports that adopted oral quinine or quinine plus clindamycin for completing of initial parenteral quinine for complicated malaria in first trimester was 12/35 (34.3%) and 3/35 (8.6%), respectively (Table 2).

### Complicated falciparum malaria during all trimesters

Intravenous artesunate has been approved by 18/35 (51.4%) and 31/35 (88.6%) of guidelines/reports for treating severe malaria in early (first trimester) and late (second and third trimesters) of pregnancy, respectively (Table 2).

### Malaria chemoprophylaxis in pregnancy in moderate-to-severe areas

Of the 23 guidelines/PMI reports to implement IPTp-SP strategy, emphasis on DOT was overlooked by 8 (34.8%). The standard dose of folic acid (400 µg daily) recommended by WHO Guidelines was adopted by 18/23 (78.3%) of SSA countries that implemented IPTp-SP strategy (Table 2). Of note, 17/23 (73.9%) did not include an explicit warning of adverse events potential if concomitant cotrimoxazole prophylaxis is used in HIV-positive pregnant women.

## Discussion

This review evaluated the treatment and prevention of falciparum malaria in pregnancy in 35 national guidelines/PMI-MOP reports out of 95 malaria-endemic countries as compared to WHO Guideline recommendation [1].

### Uncomplicated malaria in first trimester

The national guidelines and PMI reports of several representative SSA and GMS countries recommend quinine monotherapy for 7 days as the preferred regimen, administered under full supervision, for treating uncomplicated malaria during first trimester (Table 2). The use of quinine alone is contrary to WHO Guidelines, which strongly recommend quinine in combination with clindamycin for 7 days [1]. Although quinine at the recommended dose is considered safe in first trimester [1], quinine use in pregnant women is associated with drawbacks [17, 18]. The clearance of quinine is increased in pregnant women compared to non-pregnant women [19, 20]. Meta-analysis findings of five randomized trials in pregnant women revealed that a 7-day course of quinine monotherapy was linked with a slower rate of malaria parasite clearance and higher rate of gametocyte carrier emergence risk compared to ACT [21] or quinine plus clindamycin regimens [17, 22, 23].

The gametocidal activity of quinine against falciparum malaria has been reported to be sub-optimal to prevent transmission in endemic areas [18, 24]. Nausea and vomiting often associated with quinine may exacerbate morning sickness [25]. A 7-day course of oral quinine should be administered under full supervision because of poor tolerability that may compromise patient compliance resulting in treatment failure [17, 26–29]. Nonetheless, the reviewed data suggest a trend towards quinine monotherapy by two-thirds of national guidelines, although robust evidence supports the need to combine quinine with clindamycin. In real-world settings, a combination of oral quinine plus clindamycin for a 7-day treatment course has been reported to improve the therapeutic efficacy against multidrug-resistant *P. falciparum* in first trimester of pregnancy and to reduce the risk of therapeutic failure [22, 23, 26].

Moreover, this combined regimen is considered important in children and pregnant women in whom the use of doxycycline, the slow-acting partner schizonticide, is contra-indicated [26]. The explanation for quinine plus clindamycin regimen under-use is most likely the cost in endemic areas with resource constraints [22, 26]. Using International Medical Products Price Guide [30], the estimated current average cost of quinine plus clindamycin regimen (administered twice daily for 7 days) was equal to $4.05–5.95 (Table 3) in contrast to $18.5 for combined regimen [22] and $15.0 for clindamycin alone [26], reported two decades ago. These cost differences are because the estimated treatment cost was based on the supplier/buyer average price of generic clindamycin. In contrast, the treatment cost reported in previous studies [22, 26] was based on the price of proprietary clindamycin (Dalacin C). A meta-analysis of seven randomized controlled trials has affirmed that the quinine plus clindamycin regimen was safe and rarely associated with severe adverse events [23]. Perhaps neither the cost of such regimen nor adverse effects are the reasons for not recommending them by some guidelines/PMI reports. Treatment regimen complexity, including the duration, frequency of dosing, number of pills prescribed (Table 3), and poor tolerability by pregnant women with uncomplicated malaria who may require full supervision, are more likely barriers to universal endorsement of quinine plus clindamycin regimen. This controversy can be resolved by revising WHO Guidelines to include ACT as a first-line treatment option for uncomplicated malaria in first trimester as recommended by the WHO Malaria Policy Advisory Committee [31] based on growing evidence on artemisinin safety [32–35]. US-CDC updated recommendation in 2018 states that during first trimester of pregnancy falciparum malaria should be treated with the currently available options of either mefloquine or quinine plus clindamycin. However, when neither of these options is available, AL should be considered for treatment [36].

**Table 3:** Malaria in pregnancy treatment regimens and estimated costs

Anti-malarial drugs	Strength/dosage form	Median unit price^†^ (US $)	Dosage/frequency	Total number of tab/cap/vial/ampule	Total cost (US$)
Oral medications					
Clindamycin	150 mg tab/cap	0.077 bmp	4 tabs given twice daily for 7 consecutive days	56 Tap-Cap	4.3
Clindamycin	300 mg tab/cap	0.087 bmp	2 tabs given twice daily for 7 consecutive days	28 Tab-Cap	2.4
Median clindamycin price					3.4
Quinine	300 mg tab/cap	0.059 smp	2 tabs given twice daily for 7 consecutive days	28 Tab-Cap	1.65
Clindamycin + Quinine					4.05*–5.95**
Parenteral medications					
Artesunate (IV)	60 mg/vial (3 vials for 51–75 kg individual)	1.91 smp	2.4 mg/kg at 0, 12, 24, and 48 h	12 Vial	22.92
Quinine (IV)	250–300 mg/ml ampule	0.42 bmp	10 mg/kg at 0, 8, 16, and 24 h	12 ampules (including loading dose)	5.04
Artemisinin-based combinations					
Artemether + Lumefantrine (AL)	20 mg + 120 mg (SPC)^**‡**^	0.16 smp	4 tabs given at 0, 8, 24, 36, 48, and 60 h over 3 consecutive days	24 Tab-Cap	3.84
Artesunate + Amodiaquine (AS–AQ)	100 mg + 270 mg (SPC)^**‡**^	0.85 smp	2 tabs given once daily for 3 consecutive days	6 Tab	5.1
Dihydroartemisinin + Piperaquine (DHA–PPQ)	40 mg + 320 mg (SPC)^**‡**^	0.23 smp	4 tabs given once daily for 3 consecutive days	12 Tab-Cap	2.8
Medication for chemoprophylaxis in pregnancy					
Sulfadoxine + Pyrimethamine (SP)	500 mg + 25 mg (SPC)^**‡**^	0.039 smp	3 tabs given at every scheduled antenatal visit from 2nd trimester at least 1 month apart up to delivery	12 Tab-Cap^**§**^	0.47

bmp: Buyer median price; smp: Supplier median price; IV: intravenous

^†^ Calculated from international medical products price guide^30^

^‡^ Single-pill combination

^§^ Calculated as a four antenatal care visit

* Median price of Quinine + 300 mg Clindamycin combination

** Median price of Quinine + 150 mg Clindamycin combination

In some instances, the quinine dose is reduced, and clindamycin dosage may vary

### Uncomplicated and complicated malaria in second and third trimesters

In 2006, WHO [37] recommended the use of ACT, including AL, artesunate–amodiaquine (AS–AQ), and artesunate–mefloquine (AS–MQ), to treat uncomplicated falciparum malaria in second and third trimesters, but not during first trimester, until safety data becomes available. Except for dihydroartemisinin–piperaquine (DHA–PPQ) combination, ACT was considered first-line treatment in second and third trimesters [14]. In 2015, WHO recommended the use of ACT, including DHA–PPQ, as first-line treatment in second and third trimesters [1]. More recent data from SSA and many GMS countries confirm that exposure to ACT (AL or artemisinin derivatives) were not associated with adverse pregnancy outcomes during first trimester [32–36, 38] as well as in second and third trimesters [25, 32, 36, 38–41]. Adverse effects of AL, such as asthenia, poor appetite, dizziness, nausea, and vomiting, occur significantly less often with AL compared to other ACT [42]. In addition to WHO recommendations [1], several guidelines of non-endemic-malaria countries have adopted AL as the preferred treatment option for falciparum malaria in second and third trimesters [36, 43–46].

Among artemisinin-based combinations recommended by the WHO [1], AL as first-line choice has been widely recommended by approximately three-quarters of guidelines/PMI reports for treatment of uncomplicated and complicated falciparum malaria in second and third trimesters, both in endemic and non-endemic regions (Table 2). This finding harmonizes with the recent World Malaria Report for treating uncomplicated confirmed falciparum malaria in the general population [15]. As first-line treatment, AL is recommended in 24 of 46 (52%) SSA countries and 11 of 20 (55%) Southeast Asian and Western Pacific countries, respectively [15]. The extensive use of AL is due to efficacy, safety, moderate cost (compared to other ACT; Table 3); availability as a flavoured, dispersible, paediatric formulation is a distinct advantage for infants, toddlers and preschool children [47]. However, in countries, such as Cambodia where ACT resistance is a problem, national guidelines for first-line therapy need to be updated regularly because of emergent AS–MQ resistance and treatment failure. A recent randomized clinical trial carried out in Bangladesh, Cambodia, Laos, Burma, and the Democratic Republic of Congo has affirmed the safety of triple ACT, such as DHA–PPQ plus mefloquine versus AL plus amodiaquine, might provide effective treatment and delay emergence of multidrug anti-malarial resistance [48].

Sub-optimal exposure to lumefantrine, the partner component of artemether in AL (SPC) that may compromise therapeutic efficacy, is a concern with a 3-day course of AL in treating malaria during late pregnancy [49, 50]. The plasma concentrations of lumefantrine treatment were approximately 20% less in pregnant than in non-pregnant women [50], due to pregnancy-related physiological changes in drug absorption, and biotransformation (primarily due to altered hepatic cytochrome P450 isoenzymes), and increased apparent volume of drug distribution [11]. Prolonging AL treatment duration from the recommended twice daily for 3-days regimen to the extended 5-days regimen (twice daily for 5 days) was associated with acceptable tolerability and adequate safety. It enhanced overall lumefantrine exposure during late trimesters of pregnancy [49, 50] and among the general population in artemisinin resistance-emergent regions [51]. Further clinical trials evaluating the 5-days extended AL regimen for malaria treatment are awaited [50].

The oral bioavailability of lumefantrine, a lipophilic drug, is enhanced if it is administered along with milk or fat-rich meal [1]. Fat-rich food increased the bioavailability of artemether and lumefantrine by threefold and sixfold, respectively [52]. The therapeutic concentration of lumefantrine must be sustained for four 48-h asexual life cycles of *P. falciparum* and to be at least twice the minimum parasiticidal concentration on day 7 after the start of treatment in order to optimize the cure rates and to reduce the emergence of resistance and early stages of gametocyte development [53]. Ingestion of a fat-rich diet to enhance AL oral bioavailability has received little attention by most of the national guidelines/PMI reports (Table 2). Except for Ethiopia [54], Saudi Arabia [45], South Africa [46], and Sri Lanka [55], the vast majority of national guidelines have not addressed this crucial issue, an essential factor for ensuring lumefantrine plasma concentrations up to day 7 of treatment.

### Complicated malaria during any trimesters of pregnancy

Severe malaria in pregnancy needs a fast-acting and very effective treatment to save the mother’s life so that the risk/benefit equation overcomes any concerns about the safety of parenteral AS. Artesunate is first-line anti-malarial drug recommended for treatment of complicated (severe) malaria in all trimesters of pregnancy by international guidelines [1, 56], based on evidence from various studies [17, 18, 28]. In contrast to quinine, artesunate had shorter parasite clearance time and lower gametocyte carriage rate in falciparum malaria [57]. Artesunate is well tolerated [58] and has a better safety profile on the risk of miscarriage [33, 58] or significant congenital malformations [33] if administered during pregnancy compared to quinine. Unlike quinine, artesunate does not require an initial loading dose in severe malaria [1]. Moreover, quinine is associated with a higher risk of hypoglycaemia compared to artesunate, particularly in second and third trimesters, which may be refractory to intravenous glucose and may even be fatal [18, 28].

Whereas 18 of 35 (51.4%) of the reviewed guidelines/PMI reports have adhered to international guidelines regarding intravenous artesunate as a first-line option for treating complicated malaria in first trimester of pregnancy [1, 56], almost all guidelines have adopted intravenous artesunate for treating complicated malaria in the second and third trimesters. The guidelines for Benin [54] and Sudan [59] recommend either parenteral quinine or artesunate whereas in Cote d’Ivoire [54] parenteral quinine is recommended to treat complicated malaria during second and third trimesters. Paradoxically, however, in Cote d’Ivoire [54] parenteral artesunate is recommended for complicated malaria in general population. However, severe and refractory quinine-induced hypoglycaemia is more likely to occur in late than early trimesters [1], unless parenteral quinine is administered concomitantly along with glucose. The reluctance of some guidelines to recommend artesunate for treating complicated malaria in the first trimester can be attributed to safety risk controversy over artesunate; quinine during early trimester is associated with potentially less hypoglycaemic risk than during late trimesters [1]. Moreover, drug cost seems to be a deterrent for not recommending artesunate, since intravenous artesunate per treatment course costs $22.92 compared to $5.04 for intravenous quinine (Table 3). High drug cost is an impediment, especially in countries with limited healthcare budgets.

### Malaria chemoprevention in pregnancy

Based on robust evidence, WHO [8, 9] has proposed the IPTp strategy to mitigate adverse effects of malaria on maternal and fetal outcomes. IPTp should be implemented in malaria-endemic regions with high (stable) transmission as in Africa, where falciparum malaria is more prevalent. The IPTp policy requires that SP is administered to pregnant women (regardless of whether malaria parasites are identified in peripheral blood film or otherwise) at each scheduled antenatal visits starting as early as possible at the beginning of the second trimester around 13th week of gestation. Subsequent doses are to be administered at monthly intervals until delivery. The IPTp-SP strategy should be implemented as DOT of three SPC tablets (each tablet containing 500 mg/25 mg SP) [1, 8], administered by attending healthcare personnel as an integral component of antenatal care. DOT requirement of IPTp-SP policy is a vital strategy to ensure that the full preventive dose (i.e., three SPC of SP) is indeed ingested. Among 23 African countries that are required to implement IPTp-SP, the DOT requirement has not been explicitly stated in 8 (37.8%) national guidelines/reports, including that of Angola [54], Benin [54], Ghana [54], Liberia [54], Madagascar [54], Sierra Leone [54], Zambia [54], and Somalia [60] (Table 2). Although several guidelines adopt the DOT approach, its implementation in real-world practice is uncertain. Recently, poor adherence to DOT has been reported in Kenya, Nigeria and Tanzania, although DOT has been explicit in malaria guidelines of these countries [61]. National malaria plans of Ethiopia [54], South Africa [46] and Sudan [59] do not include IPTp-SP strategy as a part of their national malaria plans because of the relatively low intensity of malaria transmission in these countries, and in Rwanda due to increased parasite resistance to SP [54]. Antenatal healthcare providers should carefully adhere to the recommended DOT policy to ensure that the IPTp-SP strategy is effectively implemented. It is worth noting that WHO has not recommended IPTp strategy in countries with low transmission or high SP resistance, which is a gap in care for pregnant women, and involves other strategies, such as frequent testing and treating.

The requirement to administer daily dose of folic acid in the IPTp-SP strategy merits further attention. The recommended dose of folic acid in early pregnancy (400 µg daily) along with iron is for prevention of maternal anaemia. However, women with high risk or family history of neural tube defects should receive 5000 µg (5 mg) daily instead, to prevent neural tube defects, ideally administered before pregnancy, but if in first trimester when it would not conflict with IPTp since it is not recommended in the first trimester. The therapeutic dilemma over folic acid is among those women who have severe anaemia, especially associated with risk of severe malaria, who may require iron and higher dose of folic acid in excess of the usual recommended dose; WHO Guidelines are not clear on how this should be managed. According to WHO recommendations [8], folic acid supplement at a dose of 400 µg daily should be administered to pregnant women on IPTp-SP since it assures desired benefits without compromising IPTp-SP effectiveness. However, dose ≥ 5000 µg per day should be avoided because it antagonizes the anti-malarial efficacy of folate antagonists (SP) by pharmacodynamic antagonism [8, 62]. Almost all guidelines/PMI reports that adopted IPTp-SP strategy in their national guidelines have adhered to the WHO-recommended dose of folic acid, except for Angola [54], Cameroon [54] and Mozambique [54] PMI reports, which advocate ≥ 1000 µg daily. The guidelines need to be clear on moderate and severe anaemia, which is common in pregnant women especially in Asia where higher doses of folic acid may be justified along with other interventions, such as de-worming and vitamin A supplementation.

Falciparum malaria in pregnancy causes several harmful severe effects on maternal and fetal outcomes. WHO IPTp-SP strategy is recommended to prevent or mitigate malaria-related outcomes [8]. In pregnant women, HIV infection is associated with a significant increase in the prevalence of malaria [63, 64]. In HIV-positive pregnant women, cotrimoxazole prophylaxis is required to prevent *Pneumocystis jirovecii* opportunistic infection [65]. Of note, IPTp-SP strategy is inappropriate in HIV-infected pregnant women who are already on cotrimoxazole prophylaxis to avoid the exacerbation risk of sulfonamide-related adverse effects, especially severe cutaneous reaction [66]. WHO IPTp-SP strategy remains valid even in areas where quintuple mutations linked to SP resistance are prevalent in *P. falciparum* [9]. However, a recent meta-analysis showed that IPTp-SP might not be effective in areas where the prevalence of sextuple mutation is common, and alternative strategies to replace IPTp-SP is a pressing research priority to control malaria in pregnancy [67]. Therefore, it may be arguable whether WHO IPTp-SP strategy remains valid. DHA–PPQ is a valuable alternative to replace SP in IPTp [68], and more recent meta-analysis data have confirmed that previous concerns about repolarization-related cardiotoxicity need not limit its use for the prevention and treatment of malaria [69]. However, concurrent use of cotrimoxazole and DHA–PPQ is best avoided. Moreover, cotrimoxazole prophylaxis has been demonstrated to be non-inferior to WHO IPTp-SP strategy in pregnant women with HIV regarding infant mortality, LBW, placental malaria, maternal death, and treatment-limiting adverse events [70]. Despite the clinical importance of IPTp-SP contra-indication in such patients, this therapeutic issue has received scant attention in several guidelines. Seventeen of 23 (73.9%) PMI reports stating IPTp-SP policy, do not provide guidance on adverse consequences of drug–drug interactions between SP and cotrimoxazole (Table 2). Although more than one million HIV-positive women at risk of contracting malaria become pregnant every year in SSA countries, such as Kenya, Malawi, Mozambique, Tanzania, and Zambia [71], the national guidelines of these countries have overlooked and never addressed this important public health issue (Table 2).

### Limitations of this review

First, non-inclusion of several national guidelines of Eastern Mediterranean and Asian countries known to be endemic with malaria such as Yemen, Syria, Iran, Iraq, Pakistan, and Afghanistan; second, non-inclusion of Francophone countries in North Africa, and Hispanophone and Lusophone countries of PAHO in Central and South America eliminated the national guidelines of many countries with high malaria prevalence.

## Conclusion

This review revealed that most of the revised national guidelines/PMI-MOP reports are non-compliant to WHO Guidelines recommendations. Two-third of the revised guidelines have adopted oral quinine monotherapy to treat uncomplicated malaria during first trimester, instead of quinine plus clindamycin combination recommended by WHO Guidelines. Among ACT, three-quarters of guidelines for uncomplicated and complicated malaria recommend AL during second and third trimesters. A few of the guidelines continue to recommend intravenous quinine instead of artesunate for treating complicated malaria infections in late pregnancy in spite of potential high risk of refractory and fatal hypoglycaemia. Some countries have been slow to adopt parenteral artesunate at all, unrelated to specific concerns around safety in pregnancy.

Contrary to WHO recommendations, half of the guidelines recommend intravenous quinine instead of artesunate for treating complicated malaria in first trimester, most likely due to drug cost considerations. As a component of IPTp-SP strategy, DOT has been overlooked by one-third of the guidelines that recommend IPTp-SP policy. Three-quarters of the revised guidelines have overlooked the contra-indication of IPTp-SP implementation in HIV-co-infected pregnant women receiving cotrimoxazole prophylaxis. These lacunae in the guidelines of several countries must be addressed by policymakers to comply with current evidence-based international guidelines.

## Data Availability

Not applicable.

## Abbreviations

ACT: Artemisinin-based combination therapy
AL: Artemether–lumefantrine
AS: Artesunate
AS–AQ: Artesunate–amodiaquine
AS–MQ: Artesunate–mefloquine
CSA: Chondroitin sulfate-A
DHA–PPQ: Dihydroartemisinin–piperaquine
DOT: Directly observed therapy
GMS: Greater Mekong Sub-region
HIV: Human immunodeficiency virus
IPTp-SP: Intermittent preventive treatment in pregnancy with sulfadoxine-pyrimethamine
LBW: Low birth weight
MOP: Malaria Operational Plans
MQ: Mefloquine
PAHO: Pan-American Health Organization
PMI: President’s Malaria Initiatives
RBCs: Red blood cells
SP: Sulfadoxine-pyrimethamine
SPC: Single-pill combination
SSA: Sub-Saharan Africa
US-CDC: US Centers for Disease Control and Prevention
WHO: World Health Organization

## References

[1] WHO. Guidelines for the treatment of malaria. Third edition, 2015. Geneva, World Health Organization, 2015. https://www.who.int/malaria/publications/atoz/9789241549127/en/. Accessed 15 Jun 2020.

[2] Population reference bureau (PRB). Malaria Continues to Threaten Pregnant Women and Children. 2001. https://www.prb.org/malariacontinuestothreatenpregnantwomenandchildren/. Accessed 8 Jun 2020.

[3] McGregor IA, Wilson ME, Billewicz WZ (1983). Malaria infection of the placenta in The Gambia, West Africa; its incidence and relationship to stillbirth, birthweight and placental weight. Trans R Soc Trop Med Hyg.

[4] Duffy PE, Fried M (2005). Malaria in the pregnant woman. Curr Top Microbiol Immunol.

[5] Sharma L, Shukla G (2017). Placental malaria: a new insight into the pathophysiology. Front Med (Lausanne)..

[6] Conroy AL, McDonald CR, Kain KC (2012). Malaria in pregnancy: diagnosing infection and identifying fetal risk. Expert Rev Anti Infect Ther..

[7] Fried M, Muehlenbachs A, Duffy PE (2012). Diagnosing malaria in pregnancy: an update. Expert Rev Anti Infect Ther..

[8] WHO. Policy brief for the implementation of intermittent preventive treatment of malaria in pregnancy using sulfadoxine-pyrimethamine (IPTp-SP). Geneva, World Health Organization, 2013. https://www.who.int/malaria/publications/atoz/policy_brief_iptp_sp_policy_recommendation/en/. Accessed 15 Jun 2020.

[9] WHO. Malaria: intermittent preventive treatment in pregnancy (IPTp). Last update: 21 October 2019. Geneva, World Health Organization. https://www.who.int/malaria/areas/preventive_therapies/pregnancy/en/. Accessed 8 Jun 2020.

[10] Nosten F, ter Kuile F, Maelankirri L, Decludt B, White NJ (1991). Malaria during pregnancy in an area of unstable endemicity. Trans R Soc Trop Med Hyg.

[11] Mosha D, Guidi M, Mwingira F, Abdulla S, Mercier T, Decosterd LA (2014). Population pharmacokinetics and clinical response for artemether-lumefantrine in pregnant and nonpregnant women with uncomplicated *Plasmodium falciparum* malaria in Tanzania. Antimicrob Agents Chemother.

[12] Rijken MJ, McGready R, Boel ME, Poespoprodjo R, Singh N, Syafruddin D (2012). Malaria in pregnancy in the Asia-Pacific region. Lancet Infect Dis..

[13] Herchline TE, Bronze MS. Malaria: Practice essentials, background, etiology. Medscape reference. https://emedicine.medscape.com/article/221134-overview. Accessed 4 Jun 2020.

[14] WHO. Guidelines for the treatment of malaria, 2nd Edn. Geneva, World Health Organization, 2010. https://www.ncbi.nlm.nih.gov/books/NBK254223/. Accessed 5 Jun 2020.

[15] WHO. World malaria report 2019. Geneva, World Health Organization, 2019. https://www.who.int/publications-detail/world-malaria-report-2019. Accessed 8 Jun 2020.

[16] Center for disease control and prevention (CDC). President’s Malaria Initiative (PMI). https://www.cdc.gov/malaria/malaria_worldwide/cdc_activities/pmi.html. Accessed 8 Jun 2020.

[17] Saito M, Gilder ME, McGready R, Nosten F (2018). Antimalarial drugs for treating and preventing malaria in pregnant and lactating women. Expert Opin Drug Saf..

[18] Achan J, Talisuna AO, Erhart A, Yeka A, Tibenderana JK, Baliraine FN (2011). Quinine, an old anti-malarial drug in a modern world: role in the treatment of malaria. Malar J..

[19] Supanaranond W, Davis TM, Pukrittayakamee S, Silamut K, Karbwang J, Molunto P (1991). Disposition of oral quinine in acute falciparum malaria. Eur J Clin Pharmacol.

[20] Tarning J, Kloprogge F, Dhorda M, Jullien V, Nosten F, White NJ (2013). Pharmacokinetic properties of artemether, dihydroartemisinin, lumefantrine, and quinine in pregnant women with uncomplicated *Plasmodium falciparum* malaria in Uganda. Antimicrob Agents Chemother.

[21] Saito M, Gilder ME, Nosten F, McGready R, Guérin PJ (2017). Systematic literature review and meta-analysis of the efficacy of artemisinin-based and quinine-based treatments for uncomplicated falciparum malaria in pregnancy: methodological challenges. Malar J..

[22] McGready R, Cho T, Villegas L, Brockman A, van Vugt M (2001). Randomized comparison of quinine-clindamycin versus artesunate in the treatment of falciparum malaria in pregnancy. Trans R Soc Trop Med Hyg.

[23] Obonyo CO, Juma EA (2012). Clindamycin plus quinine for treating uncomplicated falciparum malaria: a systematic review and meta-analysis. Malar J..

[24] Yeka A, Achan J, D’Alessandro U, Talisuna AO (2009). Quinine monotherapy for treating uncomplicated malaria in the era of artemisinin-based combination therapy: an appropriate public health policy?. Lancet Infect Dis..

[25] Piola P, Nabasumba C, Turyakira E, Dhorda M, Lindegardh N, Nyehangane D (2010). Efficacy and safety of artemether-lumefantrine compared with quinine in pregnant women with uncomplicated *Plasmodium falciparum* malaria: an open-label, randomised, non-inferiority trial. Lancet Infect Dis..

[26] Pukrittayakamee S, Chantra A, Vanijanonta S, Clemens R, Looareesuwan S, White NJ (2000). Therapeutic responses to quinine and clindamycin in multidrug-resistant falciparum malaria. Antimicrob Agents Chemother.

[27] Adegnika AA, Breitling LP, Agnandji ST, Chai SK, Schütte D, Oyakhirome S (2005). Effectiveness of quinine monotherapy for the treatment of *Plasmodium falciparum* infection in pregnant women in Lambaréné, Gabon. Am J Trop Med Hyg..

[28] Kovacs Stephanie D, Rijken Marcus J, Stergachis Andy (2015). Treating severe malaria in pregnancy: a review of the evidence. Drug Saf.

[29] Nosten F, ter Kuile F, Thwai KL, Maelankirri L, White NJ (1993). Spiramycin does not potentiate quinine treatment of falciparum malaria in pregnancy. Trans R Soc Trop Med Hyg.

[30] Management Sciences for Health (MSH)/WHO. International Medical Products Price Guide. 2015 edition. https://www.msh.org/sites/default/files/msh-2015-international-medical-products-price-guide.pdf. Accessed 7 Jun 2020.

[31] WHO Malaria Policy Advisory Committee and Secretariat (2016). Malaria Policy Advisory Committee to the WHO: conclusions and recommendations of eighth biannual meeting (September 2015). Malar J..

[32] Saito M, Mansoor R, Kennon K, Anvikar AR, Ashley EA, Chandramohan D (2020). Efficacy and tolerability of artemisinin-based and quinine-based treatments for uncomplicated falciparum malaria in pregnancy: a systematic review and individual patient data meta-analysis. Lancet Infect Dis..

[33] Moore KA, Simpson JA, Paw MK, Pimanpanarak M, Wiladphaingern J, Rijken MJ (2016). Safety of artemisinins in first trimester of prospectively followed pregnancies: an observational study. Lancet Infect Dis..

[34] Manyando C, Njunju EM, Virtanen M, Hamed K, Gomes M, Van Geertruyden JP (2015). Exposure to artemether-lumefantrine (Coartem) in first trimester pregnancy in an observational study in Zambia. Malar J..

[35] Dellicour S, Sevene E, McGready R, Tinto H, Mosha D, Manyando C (2017). First-trimester artemisinin derivatives and quinine treatments and the risk of adverse pregnancy outcomes in Africa and Asia: a meta-analysis of observational studies. PLoS Med..

[36] Ballard SB, Salinger A, Arguin PM, Desai M, Tan KR (2018). Updated CDC recommendations for using artemether-lumefantrine for the treatment of uncomplicated malaria in pregnant women in the United States. MMWR..

[37] WHO. Assessment of the safety of artemisinin compounds in pregnancy. Report of two joint informal consultations convened. Geneva, World Health Organization, 2006. https://apps.who.int/iris/bitstream/handle/10665/43797/9789241596114_eng.pdf;jsessionid=C1B9576EAE5B24BB942FA133F84C1309?sequence=1. Accessed 8 Jun 2020.

[38] D’Alessandro U, Hill J, Tarning J, Pell C, Webster J, Gutman J (2018). Treatment of uncomplicated and severe malaria during pregnancy. Lancet Infect Dis..

[39] Burger RJ, van Eijk AM, Bussink M, Hill J, Ter Kuile FO (2015). Artemisinin-based combination therapy versus quinine or other combinations for treatment of uncomplicated *Plasmodium falciparum* malaria in the second and third trimester of pregnancy: a systematic review and meta-analysis. Open Forum Infect Dis..

[40] Kovacs SD, van Eijk AM, Sevene E, Sevene E, Dellicour S, Weiss NS (2016). The safety of artemisinin derivatives for the treatment of malaria in the 2nd or 3rd trimester of pregnancy: a systematic review and meta-analysis. PLoS ONE.

[41] Saito M, Mansoor R, Kennon K, Anvikar AR, Ashley EA, Chandramohan D (2020). Pregnancy outcomes and risk of placental malaria after artemisinin-based and quinine-based treatment for uncomplicated falciparum malaria in pregnancy: a WorldWide Antimalarial Resistance Network systematic review and individual patient data meta-analysis. BMC Med..

[42] The PREGACT Study Group (2016). Four artemisinin-based treatments in African pregnant women with malaria. N Engl J Med.

[43] Public Health England. Guidelines for malaria prevention in travellers from the UK 2019. https://assets.publishing.service.gov.uk/government/uploads/system/uploads/attachment_data/file/833506/ACMP_Guidelines.pdf. Accessed 9 Jun 2020.

[44] Askling HH, Bruneel F, Burchard G, Castelli F, Chiodini PL, Grobusch MP (2012). Management of imported malaria in Europe. Malar J..

[45] Ministry of Health, Saudi Arabia. National Malaria Drug Policy. 3rd edition 2018. https://www.moh.gov.sa/Ministry/About/Health%20Policies/029.pdf. Accessed 8 Jun 2020.

[46] Department of Health, Republic of South Africa. National Guidelines for the Treatment of Malaria, South Africa 2019. https://www.nicd.ac.za/wp-content/uploads/2017/03/National-Guidelines-for-Malaria-Treatment-SEPTEMBER-2019-Update-WITH-FRONT.pdf. Accessed 8 Jun 2020.

[47] Ogutu B (2013). Artemether and lumefantrine for the treatment of uncomplicated *Plasmodium falciparum* malaria in sub-Saharan Africa. Expert Opin Pharmacother.

[48] van der Pluijm RW, Tripura R, Hoglund RM, Phyo AP, Lek D, Islam A (2020). Triple artemisinin-based combination therapies versus artemisinin-based combination therapies for uncomplicated *Plasmodium falciparum* malaria: a multicentre, open-label, randomised clinical trial. Lancet.

[49] Onyamboko MA, Hoglund RM, Lee SJ, Kabedi C, Kayembe D, Badjanga BB (2020). A randomised controlled trial of 3 versus 5 days artemether-lumefantrine regimen for uncomplicated *Plasmodium falciparum* treatment in pregnancy in Africa. Antimicrob Agents Chemother.

[50] Kloprogge F, Workman L, Borrmann S, Tékété M, Lefèvre G, Hamed K (2018). Artemether–lumefantrine dosing for malaria treatment in young children and pregnant women: a pharmacokinetic-pharmacodynamic meta-analysis. PLoS Med..

[51] Tun KM, Jeeyapant A, Myint AH, Kyaw ZT, Dhorda M, Mukaka M (2018). Effectiveness and safety of 3 and 5 day courses of artemether-lumefantrine for the treatment of uncomplicated falciparum malaria in an area of emerging artemisinin resistance in Myanmar. Malar J..

[52] Lefèvre G, Thomsen MS (2012). Clinical pharmacokinetics of artemether and lumefantrine (Riamet^®^). Clin Drug Invest..

[53] WHO. Methods and techniques for assessing exposure to antimalarial drugs in clinical field studies. Informal consultation organized by the world health organization with the technical support of the worldwide antimalarial resistance network. 22–24 February, 2010 Bangkok, Thailand. https://apps.who.int/iris/bitstream/handle/10665/44653/9789241502061_eng.pdf?sequence=1&isAllowed=y. Accessed 3 Jun 2020.

[54] PMI. President’s Malaria Initiative: Fight malaria and saving life. Resource library FY 2019. https://www.pmi.gov/resource-library/mops/fy-2019. Accessed 8 Jun 2020.

[55] General Circular No: 02-112/2014. Department of Health Services, Colombo. Guidelines on malaria chemotherapy and management of patients with malaria. http://amc.health.gov.lk/Circulars/Treatment-guidelines_Malaria.pdf. Accessed 11 Jun 2020.

[56] CDC. Guidelines for treatment of malaria in the United States, 2019. https://www.cdc.gov/malaria/diagnosis_treatment/index.html. Accessed 15 Jun 2020.

[57] Pukrittayakamee S, Chotivanich K, Chantra A, Clemens R, Looareesuwan S, White NJ (2004). Activities of artesunate and primaquine against asexual- and sexual-stage parasites in falciparum malaria. Antimicrob Agents Chemother.

[58] McGready R, Lee SJ, Wiladphaingern J, Ashley EA, Rijken MJ, Boel M (2012). Adverse effects of falciparum and vivax malaria and the safety of antimalarial treatment in early pregnancy: a population-based study. Lancet Infect Dis..

[59] Republic of Sudan, Federal Ministry of Health. Sudan Malaria Diagnosis and Treatment Protocol 2017. https://reliefweb.int/sites/reliefweb.int/files/resources/sudan_malaria_treatment_protocol_final.21_nov_docx.pdf. Accessed 10 Jun 2020.

[60] Guidelines for the Diagnosis and Treatment of Malaria in Somalia 2016. https://www.humanitarianresponse.info/sites/www.humanitarianresponse.info/files/documents/files/malaria_diagnosis_and_treatment_guidelines_180316.pdf. Accessed 8 Jun 2020.

[61] Amankwah S, Anto F (2019). Factors associated with uptake of intermittent preventive treatment of malaria in pregnancy: a crosss-sectional study in private health facilities in Tema metropolis, Ghana. J Trop Med..

[62] Peters PJ, Thigpen MC, Parise ME, Newman RD (2007). Safety and toxicity of sulfadoxine/pyrimethamine: implications for malaria prevention in pregnancy using intermittent preventive treatment. Drug Saf.

[63] Verhoeff FH, Brabin BJ, Hart CA, Chimsuku L, Kazembe P, Broadhead RL (1999). Increased prevalence of malaria in HIV-infected pregnant women and its implications for malaria control. Trop Med Int Health..

[64] Kwenti TE (2018). Malaria and HIV coinfection in sub-Saharan Africa: prevalence, impact, and treatment strategies. Res Rep Trop Med..

[65] WHO. Malaria in HIV/AIDS patients. Last update: 27 April 2017. https://www.who.int/malaria/areas/high_risk_groups/hiv_aids_patients/en/. Accessed 10 Jun 2020.

[66] Sevene E, González R, Menéndez C (2010). Current knowledge and challenges of antimalarial drugs for treatment and prevention in pregnancy. Expert Opin Pharmacother.

[67] van Eijk AM, Larsen DA, Kayentao K, Koshy G, Slaughter DEC, Roper C (2019). Effect of *Plasmodium falciparum* sulfadoxine-pyrimethamine resistance on the effectiveness of intermittent preventive therapy for malaria in pregnancy in Africa: a systematic review and meta-analysis. Lancet Infect Dis..

[68] Desai M, Gutman J, L’lanziva A, Otieno K, Juma E, Kariuki S (2015). Intermittent screening and treatment or intermittent preventive treatment with dihydroartemisinin–piperaquine versus intermittent preventive treatment with sulfadoxine–pyrimethamine for the control of malaria during pregnancy in western Kenya: an open-label, three-group, randomised controlled superiority trial. Lancet.

[69] Chan XHS, Win YN, Mawer LJ, Tan JY, Brugada J, White NJ (2018). Risk of sudden unexplained death after use of dihydroartemisinin–piperaquine for malaria: a systematic review and Bayesian meta-analysis. Lancet Infect Dis..

[70] Suthar AB, Vitoria MA, Nagata JM, Anglaret X, Mbori-Ngacha D, Sued O (2015). Co-trimoxazole prophylaxis in adults, including pregnant women, with HIV: a systematic review and meta-analysis. Lancet HIV..

[71] ISGlobal. Research, maternal, child and reproductive health. HIV-Positive Pregnant Women in Malaria-Endemic Zones: A Vulnerable Population. 2014. https://www.isglobal.org/en_GB/-/el-riesgo-de-ser-mujer-embarazada-vih-positiva-en-una-zona-de-malaria. Accessed 12 Jun 2020.

[72] Revised (Current) Malaria Treatment Regimen Updated Version 2016- (6th Revision). National malaria control program, Directorate General of Health Services, Ministry of Health & Family Welfare, Dhaka, Bangladesh. http://www.brac.net/program/wp-content/uploads/2018/09/National-Malaria-Treatment-Regimen-2016.pdf. Accessed 8 Jun 2020.

[73] National Institute of Malaria Research New Delhi. Guidelines for Diagnosis and Treatment of Malaria in India 2014. https://www.researchgate.net/publication/327155827_Guidelines_for_diagnosis_and_treatment_of_malaria_in_india. Accessed 4 Jan 2021.

[74] Ministry of Health Malaysia. Management: Guidelines of malaria in Malaysia. https://www.moh.gov.my/index.php/file_manager/dl_item/554756755a584a69615852686269394859584a70637942515957356b645746754c31426c626d6431636e567a595734675330567a615768686447467549435967613246335957786862694277655774706443394e515535425230564e52553555583064565355524654456c4f52564e6654305a665455464d51564a4a5156394a546c394e5155784257564e4a515335775a475978587935775a47593d. Accessed 4 Jan 2021.

